# Cheese and Its Microbes Under the Light of One Health—A Comprehensive Review

**DOI:** 10.3390/foods15111935

**Published:** 2026-05-29

**Authors:** Maria de Lurdes Enes Dapkevicius, Telma de Sousa, Maria da Graça Silveira, Patrícia Poeta, F. Xavier Malcata

**Affiliations:** 1 Institute of Agricultural and Environmental Research and Technology (IITAA), University of the Azores, 9700-042 Angra do Heroísmo, Portugal; 2 Faculty of Agricultural and Environmental Sciences, University of the Azores, 9700-042 Angra do Heroísmo, Portugal; maria.ga.silveira@uac.pt; 3 Microbiology and Antibiotic Resistance Team (MicroART), University of Trás-os-Montes and Alto Douro, 5000-801 Vila Real, Portugal; telmaslsousa@hotmail.com (T.d.S.); ppoeta@utad.pt (P.P.); 4 Department of Genetics and Biotechnology, University of Trás-os-Montes and Alto Douro, 5000-801 Vila Real, Portugal; 5 Functional Genomics and Proteomics Unit, University of Trás-os-Montes and Alto Douro, 5000-801 Vila Real, Portugal; 6 Associated Laboratory for Green Chemistry, University NOVA of Lisbon, 1099-085 Caparica, Portugal; 7 Biotechnology Center of Azores (CBA/UAc), University of the Azores, 9700-042 Angra do Heroísmo, Portugal; 8 Department of Veterinary Sciences, University of Trás-os-Montes and Alto Douro, 5000-801 Vila Real, Portugal; 9 Animal and Veterinary Research Center (CECAV), University of Trás-os-Montes and Alto Douro, 5000-801 Vila Real, Portugal; 10 Associate Laboratory of Animal and Veterinary Sciences (AL4AnimalS), 5000-801 Vila Real, Portugal; 11 LEPABE—Laboratory for Process Engineering, Environment, Biotechnology and Energy, Faculty of Engineering, University of Porto, Rua Dr. Roberto Frias, 4200-465 Porto, Portugal; fmalcata@fe.up.pt; 12 ALiCE—Associate Laboratory in Chemical Engineering, Faculty of Engineering, University of Porto, Rua Dr. Roberto Frias, 4200-465 Porto, Portugal

**Keywords:** cheese, One Health, lactic acid bacteria, *Staphylococcus aureus*, *Listeria monocytogenes*, *Salmonella*, *E. coli*, *Enterococcus*, antibiotic resistance

## Abstract

A complex, dynamic microbiota inhabits cheese and cheesemaking environments. Such microorganisms can move across the dairy chain, from the microbiomes of production animals, through humans and environments which they interact with. These interactions impact all sectors of the One Health continuum. They may contribute to disseminate beneficial bacteria that enhance cheese quality, sustainability, and consumer health, but they also promote spread of pathogenic strains, along with their genetic determinants of virulence and antibiotic resistance across the milk and cheese production chain. This comprehensive, narrative review aims at providing a current view on cheese, its microbes, their roles, genetic determinants of virulence and antibiotic resistance, their relevance within the scope of the One Health approach and proposes using enterococci as sentinel microorganisms for monitoring AMR in cheese production chains.

## 1. Introduction

Cheese is an important constituent of the human diet, and a basis for several economic activities in many parts of the world (Table 1). According to OECD [1], it is the second most consumed dairy product at global scale. Increases in the global demand for cheese have been experienced or are expected even in regions where it has not been part of the traditional diet (e.g., certain areas of Asia. The high levels of cheese consumption, particularly in Europe and North America, where both per capita intake and production volumes are greatest, emphasize the nuclear role of cheese as a major pathway for microbial exposure within the human diet. Owing to its complex production chains—involving raw or thermized milk, diverse starter and adjunct cultures, and often extended ripening under conditions allowing microbial growth, cheese constitutes both a reservoir and a vector for microorganisms spanning both beneficial taxa (e.g., technologically relevant lactic acid bacteria) and potentially deleterious populations (opportunistic and pathogenic species). Cheese-related ecosystems also provide favourable conditions for microbial interaction and genetic exchange, thereby facilitating persistence and dissemination of antimicrobial resistance genes (ARGs), as well as virulence determinants through horizontal gene transfer (HGT) mechanisms.

Knowledge on cheese microbiota is building up, especially in view of the increasing availability of culture-independent methodologies; despite such knowledge being important to promote product quality and take full advantage of its nutritional potential, one is still far from having enough information available for fully educated decision-making. This review is aimed at presenting the state-of-the-art on cheese microbiota, the corresponding microbiome, how they are impacted by cheesemaking and their interplay with human health from the point of view of the One Health framework.

## 2. Cheese as a Substrate for Microorganisms—Implicit Drivers of the Cheese Microbiota

The microbiota of a mature cheese is shaped by several biotic and abiotic factors. Cheesemakers have taken advantage of those parameters in their attempts to improve quality of the final product. Among the latter, nutrient availability/limitation, pH, and salinity/*a*_w_ play central roles in the sequence of events that support microbiota building in any given cheese variety [6,7].

In a nutshell, cheesemaking starts with the curdling of milk. The curds thus obtained then undergo maturation, crucial to development of the sensory, chemical, and physical properties of the final product. During the initial milk coagulation step, lactose is abundant, and it can serve as a substrate for microbial growth. Not all microorganisms present in milk are able to thrive on lactose. This poses a first constraint upon the microbiota of the cheese to-be. Starter lactic acid bacteria (SLAB) will quickly utilize lactose, thus decreasing availability of this substrate to other members of the curd microbiota—while altering the curd environment by decreasing its pH, and releasing antimicrobial compounds that will further influence composition of the final microbiota. The decrease in curd pH as a consequence of SLAB-mediated fermentation of the retained lactose is a central event in cheesemaking. Its extent not only holds a direct influence on cheese microbiota but also impacts the physical properties of the curd. The decrease in pH influences the water holding capacity of the casein matrix in the curd by promoting syneresis, which in turn further decreases nutrient availability and starts impacting water availability. By the end of this process, cheese becomes an inhospitable environment for many microorganisms—fermentable carbohydrates are lacking, salt concentration has increased as a result of water loss, water activity and pH are lower. Maturation temperatures differ between cheese varieties but generally lie below the optimal range for growth of mesophiles—which includes both lactic acid bacteria (LAB) and most cheese borne pathogens. Faced with these constraints, SLAB numbers start to decline and will dwindle over the maturation period, thus giving place to a new set of LAB that are better adapted to survive in the challenging environment of maturing cheese—the non-starter LAB (NSLAB). The constraints that shape cheese LAB microbiota also act upon pathogens and other types of bacteria; this leads to a decrease in their numbers or, at least, some inhibition of their growth [8]. In this respect, cheesemaking can be understood as an example of hurdle technology; its main instrumental hurdles are nutrient limitation, water availability, salt concentration, pH, and temperature, besides LAB metabolites that have accumulated throughout the process of converting milk to cheese. These barriers not only select and modulate the species and strains that survive in cheese but also impact microbial physiology. Microorganisms in cheese may, in response to said barriers, assume a physiological state known as “viable, non-cultivable”, which affects culture-dependent studies on its microbiota [6], and impacts the role of cheese as a vehicle for foodborne pathogens.

The energy substrates microorganisms resort to when growing in cheese are lactose, galactose, lactate, protein, amino acids, lipids, and free fatty acids resulting from lipolysis. The extent to which each nutrient is used, as well as the timeline of nutrient utilization differ between microorganisms and cheese varieties [9]. Nutrient availability and distribution contribute to shape the cheese microbiota and its spatial organization. At the microscopic scale, cheeses are not homogenous environments—neither from the point of view of distribution of their microbial inhabitants, nor from the point of view of nutrient distribution. Entrapped within the cheese matrix, bacteria are hardly free to move. They exist in randomly distributed bacterial aggregates (colonies), shown to be 25–250 µ apart in non-fat, model cheese experiments. Metabolic activity within the colonies is not uniform. Exponential phase cells are located primarily in the outer layers of those aggregates, while the inner core is richer in cells at later growth stages [10]. Within the colony, cells that undergo lysis as maturation progresses release additional nutrients (carbon, nitrogen) that can foster cryptic growth [11]. Presence of fat in cheese, however, leads to a two-phase matrix, with colonies associated mostly with the fat-protein interface [12,13]. Colony distance and distribution may affect nutrient diffusion. However, this is regarded as a limiting factor for microbial growth in cheese only for large molecules or for those that are bound in some way to the cheese matrix. Nutrients released from lysed SLAB provide, in these circumstances, an important source of nutrients for NSLAB growth [11].

**Figure 1:**
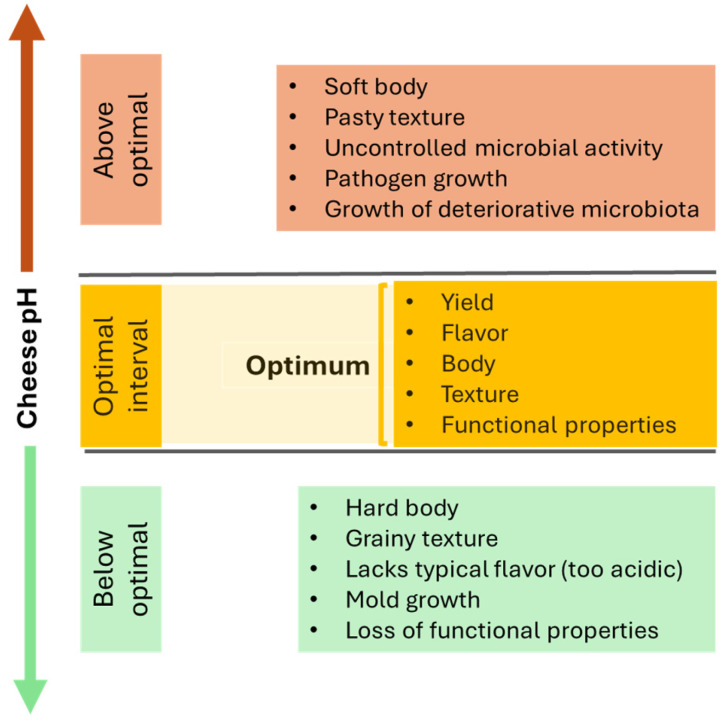
Effect of pH upon cheese quality (adapted from Bansal & Veena [15]).

The decrease in pH is a central phenomenon in cheesemaking. It has been regarded as one of the main universal drivers that shape cheese microbiome [14] but also affects many other processes and phenomena taking place at both curd formation and cheese maturation stages, e.g., rennet coagulation, whey syneresis, salt absorption, microstructure building, and rheology development [15]. The impact of pH on cheese quality is summarized in Figure 1. The decrease in pH during production and maturation exerts selective pressure, probably favouring and survival and dominance by certain species within the cheese microbiota. It has, accordingly, been demonstrated to influence the microbiota richness in several types of cheese [14]. On the other hand, process variables, such as level and method of salting, have a major impact upon pH evolution in cheese [16].

Cheese salinity, however, does not seem to play a direct role on the richness of cheese microbiota, in spite of affecting microbiota composition and growth. It also affects microbial activity and biochemical events throughout the microbial succession along maturation [14], and, ultimately, cheese quality [16]. NaCl content in most cheese varieties lies within 2—10% in terms of salt-in-moisture, which is enough to constitute a considerable barrier against bacterial growth [16]. Salinity affects, but is not the only factor implicated in, cheese *a*_w_. Further determinants are water content and the presence/concentration of other solutes. Proteolysis, a major biochemical event during cheese maturation, may be one of the major drivers in *a*_w_ decrease [17,18,19]. In addition, cheese *a*_w_ changes not only with time, but also with spatial location; it is, in fact, higher in the mass than in the crust. In the latter, moisture loss throughout maturation leads to increased concentrations of solutes and seems to be the main promoter of the decrease in water activity that usually occurs at crust level [20]. The decrease in *a*_w_ observed both at crust level and in the mass as cheese ages is an important barrier against the growth of pathogenic bacteria [20,21].

During cheese manufacture and maturation, redox potential (Eh) decreases from the positive values (between +250 and +350 mV) observed in milk [22] to negative values, that may get as low as—400 mV [23]. It also varies according to cheese locations; lower Eh values are found deeper within the mass and higher ones on the crust [23]. The development of a reducing environment within cheeses results from several factors: LAB growth, fermentation of residual lactose, and oxygen consumption/scavenging resulting from the activity of their microbiota [22,24]. This decrease affects cell metabolism and physiology [22], thus impacting the development and viability of the constituents of cheese microbiota. Eh could also affect flavour development by cheese LAB [24] (Morandi et al., 2016). The redox potential is an important determinant of the composition of the microbiota [16], discriminating and selecting obligate/facultative anaerobes within the cheese mass [24].

Besides the aforementioned major intrinsic drivers that modulate cheese microbiota, the antimicrobial systems in milk may also play a role in shaping the microbial composition and activity of cheeses. For instance, such antimicrobial agents as lactoferrin, the lactoperoxidase system, lysozyme, immunoglobulins, and some of the fatty acids present in milk may afford protection against pathogen growth. However, it must be noted that these agents can be inactivated, to a greater or lesser extent, by the heat treatments (e.g., pasteurization, thermisation) applied to milk prior to its transformation in cheese [25].

## 3. Extrinsic Drivers and the Cheese Microbiota

### 3.1. Impact of Cheesemaking Conditions on the Cheese Microbiota

**Figure 2:**
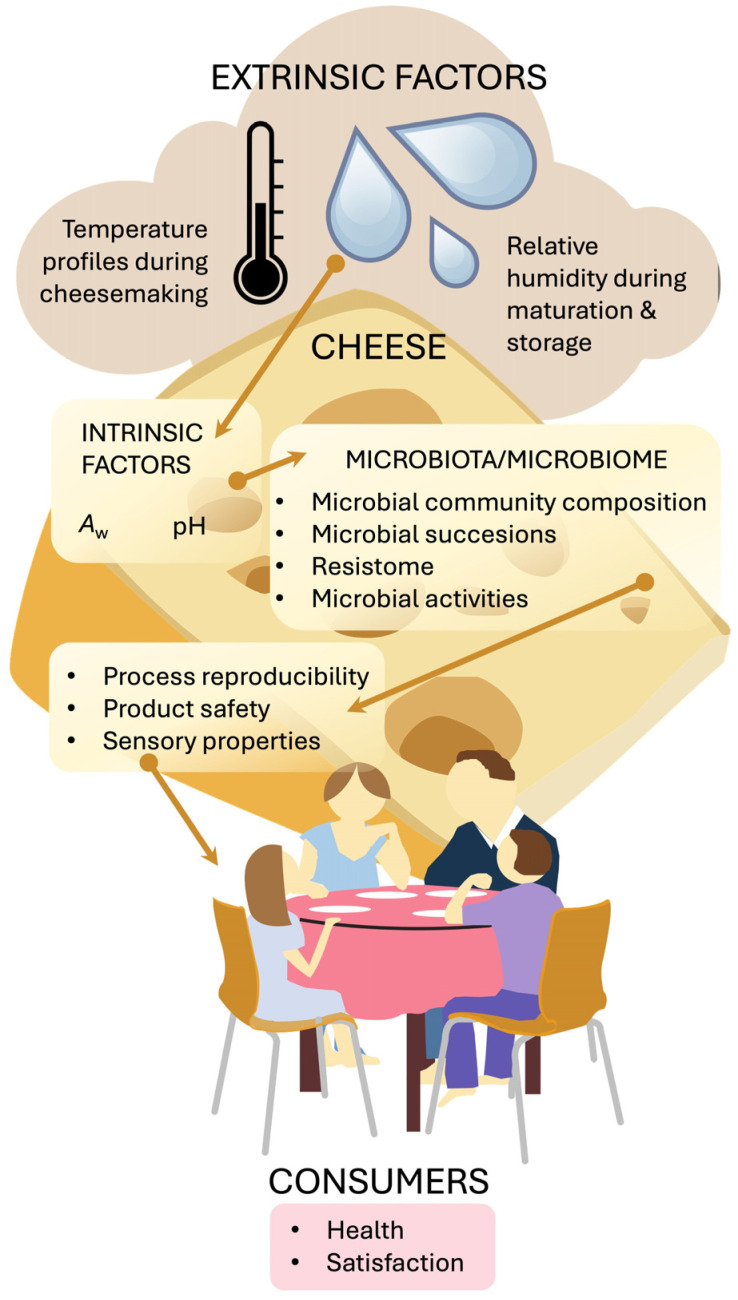
Impact of extrinsic factors on the interplay between cheese microbiota, microbiome and consumers.

As Figure 2 shows, cheesemaking is governed by a complex interplay of extrinsic factors, among which temperature and relative humidity (RH) during ripening are particularly critical in shaping cheese microbiota and their metabolic activities [26,27]. During maturation, temperature directly influences growth kinetics of both starter and non-starter microorganisms, by modulating enzymatic processes such as proteolysis and lipolysis that underpin flavour and texture development [28,29]. Higher ripening temperatures generally accelerate microbial metabolism and enzymatic activity, promoting faster proliferation of NSLAB and secondary microbiota, whereas lower temperatures slow down microbial growth and extend ripening time [28,29]. In addition, temperature gradients within the cheese matrix can generate microenvironments that select for distinct microbial populations and thus contribute to spatial heterogeneity in community structure [26,30].

Relative humidity is another critical parameter, particularly for surface-ripened and smear-ripened cheeses, where it regulates water activity at the rind and thereby governs microbial colonization and succession [27,31]. High RH values favour growth of yeasts and Gram-positive bacteria such as *Brevibacterium* and *Corynebacterium*, which are essential for rind development, deacidification, and the establishment of complex microbial consortia [26,31]. Conversely, low RH can produce excessive desiccation of the cheese surface, limiting microbial growth, altering community dynamics, and potentially causing defects in rind formation and sensory attributes [27,28]. The interaction between temperature and RH is therefore critical, as these factors jointly influence moisture loss, salt diffusion, and oxygen availability—all of which indirectly shape microbial ecology and activity [29,31].

During ripening, the combined effects of temperature and RH drive microbial succession, typically characterized by a transition from starter-dominated communities toward more diverse assemblages including NSLAB, yeasts, and filamentous fungi [26,30]. Early in maturation, lactic acid bacteria ferment residual lactose and reduce pH, thus establishing selective conditions that favour acid-tolerant and metabolically versatile microorganisms [27,29]. As ripening progresses, environmental changes—such as pH increase at the surface mediated by yeast metabolism under high RH—facilitate the growth of secondary microbiota responsible for advanced proteolysis and flavour compound production [26,31]. Temperature-dependent enzymatic activities further regulate release of peptides, amino acids, and volatile compounds, thereby linking microbial dynamics to sensory outcomes [28,29].

Deviations from optimal temperature and RH conditions can disrupt microbial balance and consequently lead to spoilage or safety concerns, including proliferation of undesirable or pathogenic microorganisms [27,31]. Inadequate control of ripening environments may also promote uneven microbial growth and metabolic activity, thus compromising both product consistency and quality [26,32]. Therefore, accurate management of extrinsic factors during cheese maturation is essential not only to guide microbial succession and biochemical transformations but also to ensure product safety, reproducibility, and desirable sensory characteristics [28,29].

### 3.2. Impact of the Unit Operations Involved in Cheesemaking on Cheese Microbiota and Antibiotic Resistance

**Figure 3:**
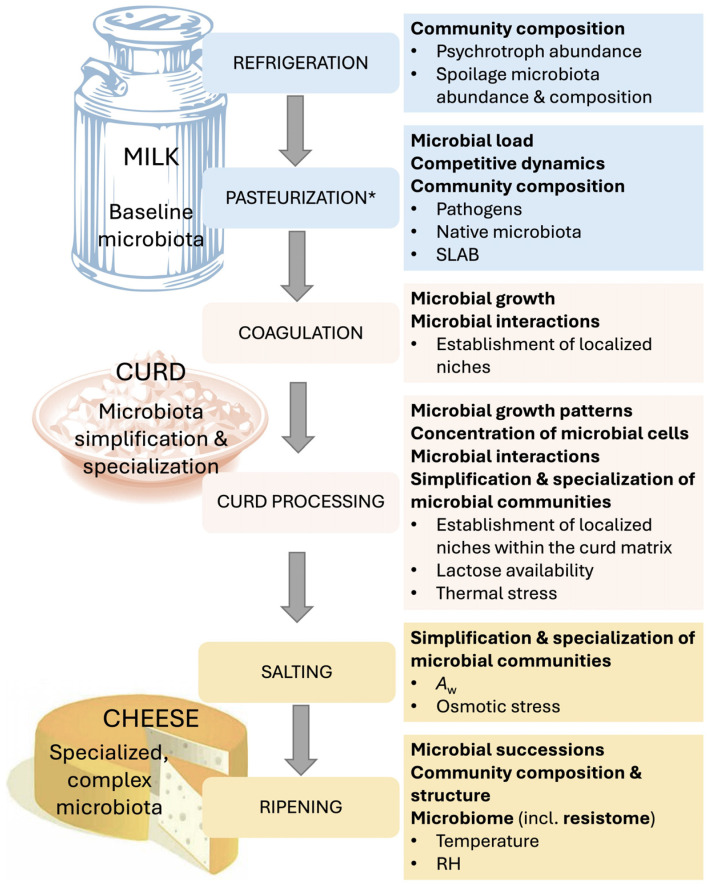
Summary of impacts of the main cheesemaking unit operations upon cheese microbiota and microbiome (* If applied).

Cheesemaking comprises a sequence of unit operations, each imposing selective pressures that shape the structure, diversity, and function of cheese microbiota from milk to ripened product (Figure 3) [33,34]. Milk handling prior to processing constitutes a critical step. Temperature control during milk storage influences the abundance of psychrotrophic and spoilage-associated taxa, thereby determining the baseline microbial community entering cheesemaking [35,36]. Pasteurization, applied during manufacture of many cheese varieties, represents a major technological intervention that reduces both microbial load and diversity in milk, while selectively eliminating pathogens and many indigenous microbial species, and thus creating ecological space for starter cultures (whenever employed) to eventually overrun the cheesemaking process [33,34]. However, heat treatment also alters the competitive dynamics within the cheese ecosystem by removing background microbiota. This can influence microbial succession trajectories and increase the susceptibility of cheeses made from pasteurized milk to post-processing contamination [28,35].

Coagulation and curd formation constitute another pivotal unit operation, during which enzymatic gelation and acidification create a structured milk protein matrix that entraps microorganisms and establishes localized niches for microbial growth and interaction [33,37]. Curd cutting, which controls moisture expulsion and curd particle size, indirectly modulates microbial ecology by influencing *a*_w_ and nutrient diffusion, thereby shaping microbial growth patterns [34,38]. Subsequent cooking or scalding steps (if applied) impose further thermal stress, selecting in favour of thermotolerant lactic acid bacteria against less heat-resistant taxa, ultimately contributing to community simplification and specialization [33,34]. Draining and whey removal also impact microbiota by reducing lactose availability and concentrating microbial cells within the curd matrix [28,37].

Salting, whether dry or achieved by brining, represents another key selective hurdle that strongly affects microbial composition by reducing *a*_w_ and exerting osmotic stress—conditions that favour salt-tolerant taxa, such as certain strains of lactic acid bacteria (a few lactobacilli, pediococci, and enterococci) and surface-ripening microorganisms [28,32]. Variations in salt concentration and distribution within the cheese matrix have been shown to influence both microbial diversity and metabolic activities, with impacts on the balance between desirable and spoilage-associated microorganisms [32,39]. Pressing and shaping operations further contribute to shape the cheese microbiota, by modifying oxygen gradients and moisture distribution, thus influencing the spatial organization of microbial communities within the cheese interior and its rind [35,37].

Ripening probably constitutes the most complex of the unit operations in cheesemaking. During this step, controlled environmental conditions (particularly temperature and RH) drive microbial successions and metabolic interactions within the cheese ecosystem [28,33,40]. As this step evolves, SLAB are gradually complemented or replaced by NS LAB, yeasts, and moulds, thus resulting in increasingly diverse and functionally specialized microbial consortia [32,37]. Environmental exposure during ripening, including contact with equipment, air, and human handlers, introduces additional microbial taxa that can colonize the cheese surface and contribute to rind development [28,35]. These surface-associated communities play a critical role in shaping sensory properties through complex microbial interactions, which include cross-feeding and competitive exclusion [32,37].

The cumulative effects of cheesemaking unit operations constitute a dynamic and highly selective process, in which sequential technological steps act as ecological filters, constraining the composition, succession, and functionality of cheese microbiota [33,34]. For this reason, technological design and process control are critical for success in cheesemaking, as even minor variations in unit operations can lead to significant shifts in microbial communities and, consequently, in cheese quality, safety, and typicity [35,36].

The unit operations encompassed by cheesemaking also impact the resistome of this product. In Europe, the artisanal production of regional cheeses remains an important component of cultural heritage. It relies largely on adventitious microorganisms in raw milk, often in the absence of defined starter cultures, which hampers control over the microbial composition of the final product [25,41]. The microbial communities present in these systems are exposed to multiple food-associated stresses during cheesemaking and storage; it is now well-established that bacterial adaptive responses to sublethal stresses (including heat, cold, acid, and osmotic stress) can promote cross-protection and modulate antimicrobial resistance phenotypes [42,43].

During milk refrigeration, microorganisms native to raw milk are exposed to cold stress. This exposure likely influences membrane composition and stress-response pathways, thereby altering susceptibility to antimicrobial agents [35,36]. Experimental evidence indicates that cold stress can enhance tolerance to antibiotics in foodborne pathogens, including *Listeria monocytogenes* and *Salmonella enterica*, with increases in minimum inhibitory concentrations (MICs) reported for several antibiotic classes [36,44]. Notably, such stress-induced phenotypes may persist after stress removal; hence, stable or semi-stable adaptations could contribute to dissemination of antibiotic resistance through the food chain [42,43]. Consequently, milk refrigeration may modulate the resistome of cheese microbiota, particularly in raw milk cheeses that bypass heat treatment, and therefore retain stress-adapted bacterial populations [25,35].

The acidification step, driven by LAB during lactose fermentation, represents another major selective pressure likely to influence antimicrobial resistance [28,43]. Exposure to sublethally acidic conditions has been shown to induce adaptive responses in pathogens such as *L. monocytogenes* and *S. enterica*, modulating antibiotic susceptibility, with effects ranging from increased to decreased resistance, depending on the compound at stake and the physiological state of the pathogen [36,42]. Acid stress can also induce membrane remodelling, efflux pump activation, and changes in intracellular ATP levels, which collectively influence antibiotic uptake and resistance phenotypes [43]. It should be emphasized that some of these adaptations may persist beyond the stress exposure period, which suggests the potential for cheesemaking conditions to select for more resilient microbial populations [42].

Salt addition during curd formation and/or brining imposes osmotic stress, another key factor influencing microbial survival and resistance dynamics in cheese [31,32]. High NaCl concentrations have been shown to induce cross-protection mechanisms in bacteria, including upregulation of efflux systems and stress-response genes, which can reduce susceptibility to antibiotics [32,43]. Studies on foodborne pathogens and commensal bacteria have indicated that osmotic stress can lead to increased MIC values for several antibiotics, thus supporting the role of salting as a driver of resistome modulation in cheese ecosystems [31,42].

Thermal treatments applied during cheesemaking, such as curd cooking or milk pasteurization, also exert strong selective pressures upon microbial communities and their resistance traits [28,35]. Sublethal heat stress can induce physiological changes that affect membrane fluidity, protein stability, and stress-response pathways, which in turn influence antibiotic susceptibility [36,42]. While some studies report increased tolerance to antibiotics following heat stress, others indicate enhanced susceptibility, reflecting species-specific responses and the complexity of stress adaptation mechanisms [43]. Importantly, these changes may be transient or persistent depending on the duration and intensity of the stress applied, as well as on the genetic background of the microorganism [42].

Conversely, lethal heat treatments such as pasteurization can reduce the abundance of antibiotic-resistant bacteria, thereby contributing to mitigate cheese resistome [35,36]. It is worth noting that antibiotic resistance has been associated with fitness costs in some bacterial populations, which may reduce their tolerance to thermal stress and confer competitive disadvantages under heat treatment conditions [43,45]. This relationship has been observed in pathogens such as *Salmonella enterica* and *Escherichia coli*, where antibiotic-resistant strains may exhibit lower thermal resistance compared to their antibiotic-susceptible counterparts [36,42].

The sequence of unit operations in cheesemaking acts as a series of selective pressures that not only shape microbial composition but also influence the structure and dynamics of the cheese resistome [35,41]. The use of sublethal preservation treatments, while effective in controlling microbial growth, may inadvertently promote emergence and persistence of stress-adapted, antibiotic-resistant populations. This highlights the need for optimized processing strategies to ensure cheese safety within the One Health framework [42,43].

Across cheese production chains, mitigation of virulent and antimicrobial-resistant (AMR) bacterial clones requires a combined strategy targeting raw material hygiene, thermal processing, and ripening ecology. At farm level, strict mastitis control, hygienic milking practices, and rapid cooling of raw milk (<4 °C) contribute to reducing initial bacterial loads and limit amplification of resistant enterococci and *Enterobacteriaceae* [46,47,48,49]. For cheeses manufactured from raw milk, a validated control system is essential, with particular emphasis on preventing contamination at the farm and during transport [47,48,50]. In cheesemaking, pasteurization remains the most effective intervention for reducing viable pathogenic and AMR bacteria in industrial cheese production. High-temperature short-time (HTST) pasteurization (72 °C for 15 s) is generally sufficient for vegetative cells, including those of enterococci, although post-pasteurization contamination must be strictly controlled through closed systems and hygienic design [51]. For thermised or raw-milk cheeses, alternative hurdles, such as competitive starter cultures and adjunct protective cultures, should be applied to suppress opportunistic AMR carriers [51]. During cheese ripening, microbial selection is strongly influenced by salt concentration, pH decline, and water activity. Brine systems represent critical reservoirs for persistent AMR bacteria and require periodic filtration, UV treatment, or thermal regeneration to prevent biofilm-associated dissemination [52,53,54,55]. Ripening conditions should be tailored to cheese type: hard cheeses benefit from longer ripening at controlled low humidity to reduce pathogen survival, while soft cheeses require stricter raw material control due to limited inhibitory barriers [32,56]. Surface-ripened cheeses necessitate regular monitoring of smear microbiota to prevent establishment of multidrug-resistant environmental strains [57]. Furthermore, integrating environmental monitoring of indicator organisms such as enterococci across processing stages enables early detection of contamination sources and supports targeted sanitation interventions. Overall, a multi-hurdle strategy combining hygienic raw milk production, validated pasteurization, and controlled ripening ecology are essential to minimize dissemination of virulent and AMR clones in cheese production systems [58].

## 4. Implicit Drivers—Properties of Microbiota Constituents and Their Interactions

**Figure 4:**
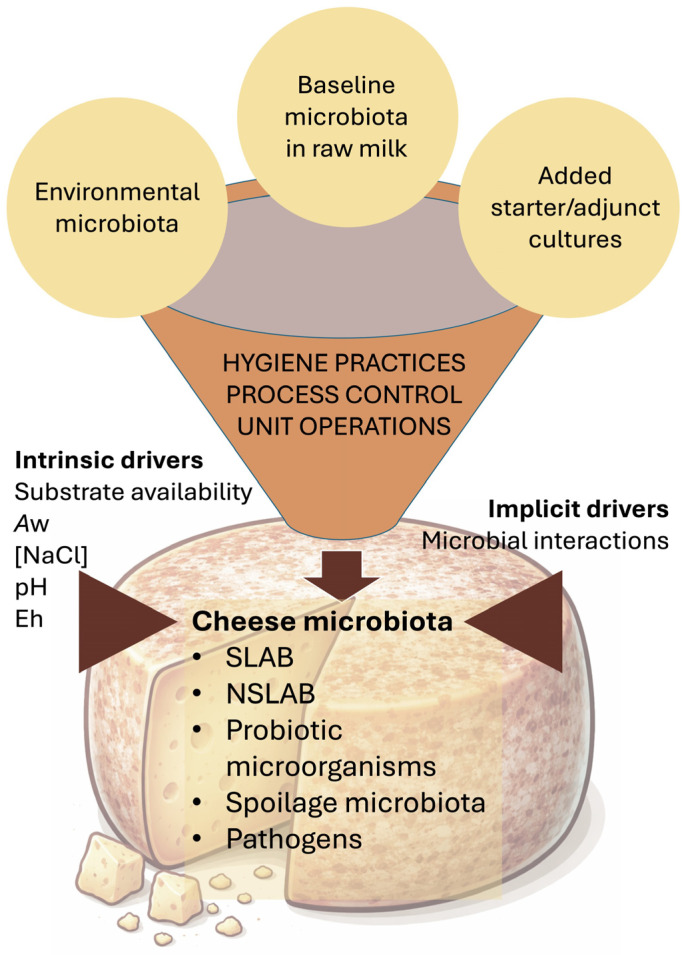
Sources of cheese microbiota, and main drivers that shape it.

Cheese microbiota is shaped by multiple drivers and originates from multiple sources (Figure 4). Raw milk represents the primary reservoir of microbial diversity in cheese, including lactic acid bacteria, spoilage organisms, and environmental contaminants [28,58,59,60]. The initial microbial quality and composition of milk strongly influence the downstream microbial dynamics, as indigenous taxa can persist throughout processing and interact with starter cultures during fermentation and ripening [28,29]. In addition to native milk microbiota, defined starter and adjunct cultures intentionally introduced during cheesemaking constitute another major source of bacteria for driving acidification and early curd formation [33]. As ripening progresses, NSLAB—often originating from raw milk or the dairy environment—become dominant and contribute significantly to the biochemical transformations typically associated with cheesemaking [28,59]. The cheesemaking environment itself (i.e., equipment, brines, air, and surfaces) acts as an important secondary source of microbiota, sometimes contributing substantially to the final community structure [28,33]. Environmental microorganisms can account for a large proportion of the microbial taxa present in certain cheeses, particularly in smear- or surface-ripened varieties [28,33]. Furthermore, human handling and processing practices are likely to introduce additional microorganisms, reinforcing the role of artisanal and facility-specific factors in shaping cheese microbiomes [28,33,61,62]. These diverse sources interact collectively and dynamically to produce complex, cheese-specific, microbial ecosystems.

Therefore, cheese microbiota is complex, dynamic, and numerous. It is dominated by LAB, particularly *Lactobacillales* [33]. The predominant genera differ according with the type of cheese. For instance, lactococci predominate in Pico cheese, an artisanal raw-milk cheese produced in the Azores archipelago (Portugal), whereas lactobacilli stand out in Protected Denomination of Origin (PDO) S. Jorge cheese, another Azorean, raw-milk cheese [63,64]. Cheese SLAB, responsible for curd acidification and for the cascade of processes that follow, can be mesophilic, coccoid LAB (mainly *Lactococcus lactis*) or thermophilic lactobacilli (*Lactobacillus helveticus*, *Lactobacillus delbrueckii*) and coccoid LAB (*Streptococcus salivarius* subsp. *thermophilus*). It is usually assumed that SLAB loose viability during early ripening, and that, from that point on, their contribution to cheese maturation arises chiefly from the release of enzymes resulting from increased permeability of their cell envelopes and, eventually, from autolysis. A subpopulation of the SLAB may, however, persist well beyond that point [65]. The NSLAB most frequently found during cheese ripening, and which contribute to flavour development, belong to the *Lacticaseibacillus* genus (*Lacticaseibacillus casei*, *Lacticaseibacillus paracasei*, and *Lacticaseibacillus rhamnosus*) [60]. NSLAB are less abundant in milk but become prevalent in cheese because they are well-adapted to cheesemaking-related physicochemical stresses (thermal, acidic, osmotic, and oxidative) [65], a realization that may have implications in terms of One Health.

LAB typically reach very high cell densities in cheese curds and throughout ripening, often becoming the dominant microbial group and thus exerting a major influence on cheese microbiota and on its interaction with consumers [28,29]. Due to these high population levels, the LAB in cheese can have a substantial effect in terms of One Health. They may act as reservoirs of relevant genetic determinants that can be transferred along the dairy chain, finding their way into the human gut microbiota [66,67]. Recent studies have demonstrated that a substantial proportion of LAB isolated from dairy products harbour acquired ARGs, including those implicated in tetracycline and macrolide resistance, thus highlighting their potential role in dissemination of antimicrobial resistance [66]. Furthermore, LAB may carry genetic determinants of virulence-associated traits, raising concerns that they may play a dual role as beneficial technological cultures and potential vectors of clinically relevant genes within the One Health continuum [57]. Given these risks, strict regulatory frameworks have been established to ensure safety of the LAB used in food production, particularly as starter and adjunct cultures [68]. In the European Union, the Qualified Presumption of Safety (QPS) system, implemented by the European Food Safety Authority (EFSA) provides a harmonized pre-assessment of microbial safety that essentially requires strains not to harbour transferable antimicrobial resistance or virulence determinants [69]. In the United States of America, many LAB used in food fermentations are classified as Generally Recognized as Safe (GRAS) [67,68]. The EU and the USA systems differ considerably in their approach to evaluating the safety of microorganisms for food applications. While the GRAS system is based on case-by-case, strain level assessment, the QPS framework is rooted in a species-level pre-evaluation followed by strain-level verification. Because the GRAS determinations can be made through self-affirmation by companies or via voluntary notification by the FDA, the USA system holds a higher degree of regulatory flexibility than its European counterpart, which is centrally managed by EFSA. These systems are important on the context of cheese LAB. The high numbers reached by LAB in cheese, combined with their genetic plasticity, underscore the need for continuous surveillance and rigorous strain selection to mitigate potential risks, while taking advantage of their technological and functional benefits [66,68].

A particular case within cheese LAB is the *Enterococcus* genus. Enterococci associated with cheese occupy a pivotal yet ambivalent position within the One Health framework, by linking food systems, animal reservoirs, human health, and environmental dissemination. These bacteria are natural components of the gastrointestinal microbiota of humans and livestock and are commonly detected as NSLAB in artisanal and industrial cheeses, where they can reach high population levels and contribute to ripening and to the cheeses’ sensory properties [41,70]. Their remarkable adaptability to the cheese environment, coupled with such metabolic activities as proteolysis and bacteriocin production, contribute to both technological relevance and ecological persistence [41]. However, cheese-derived enterococci frequently harbour genetic determinants of antimicrobial resistance and virulence factors, including multidrug resistance, biofilm formation, and genes such as *van*A, *tet*M, and *esp*. This highlights their potential to act as reservoirs and vectors of clinically relevant traits along the dairy food chain [71,72,73]. Evidence from artisanal cheeses, such as Pico cheese, where enterococci have been shown to carry both virulence determinants and antibiotic resistance traits, demonstrates their role as potential foodborne reservoirs of AMR and virulence genes, with obvious implications for food safety and public health [72]. Furthermore, enterococci may play a central role in the dissemination of antimicrobial resistance (AMR) and virulence determinants among pathogenic and non-pathogenic bacteria, due to their notable propensity to take part in recombination and horizontal gene transfer (HRGT) events [74]. The persistence of these traits across the dairy production continuum, from animal gut to raw milk, cheese matrices, and the human microbiome, underscores the risk of horizontal gene transfer and dissemination of resistance within and beyond the food chain [75]. On the other hand, enterococci can also provide useful sentinel organisms for monitoring antimicrobial resistance throughout the One Health continuum, owing to their ubiquity across human, animal, food, and environmental niches, together with their ability to provide pathways for the transmission of ARGs along these interconnected domains [73,76]. Consequently, the presence of enterococci in cheese is not only relevant for food quality and functionality but also represents a critical interface for monitoring and mitigating AMR spread within the One Health space and reinforces the need for integrated surveillance/risk assessment strategies spanning dairy production and public health systems [41,72].

**Figure 5:**
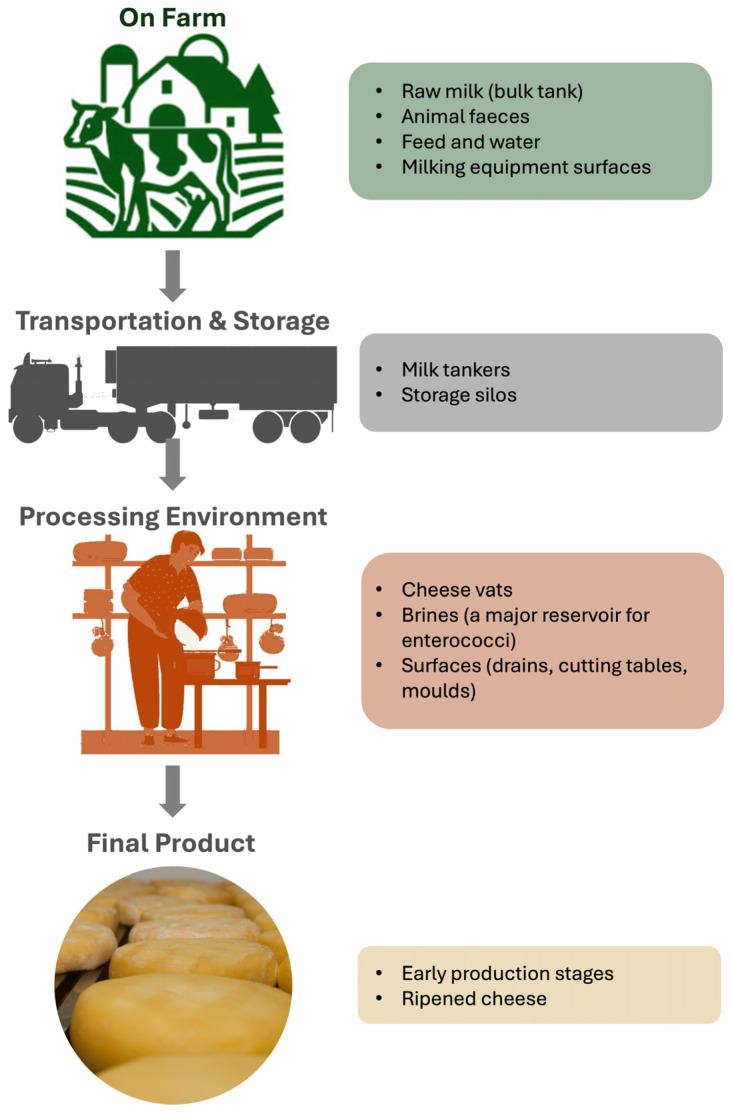
Main sampling nodes for monitoring AMR resistance in cheese production chains.

Enterococci are, thus, suitable sentinel organisms for monitoring antimicrobial resistance in cheese production chains due to their ubiquity, environmental persistence, and capacity to acquire and transfer resistance determinants. They could be used to follow AMR trends over time, to show emerging resistance patterns, to detect critical control points in the cheese production chain, and to determine resistance dissemination pathways. Surveillance should follow a farm-to-fork approach, including systematic sampling of raw milk, animal feces, feed, processing environments (e.g., vats, brines, surfaces), and cheeses at different ripening stages. Consistent, repeated sampling should be performed at defined “nodes”, located throughout the production chain (Figure 5).

Using both phenotypic and genotypic approaches is essential for accurate antimicrobial resistance (AMR) surveillance in enterococci along cheese production chains, as each method captures complementary dimensions of resistance. Phenotypic testing reflects the actual expression of resistance under defined conditions and thus provides clinically and technologically relevant information [70,77]. However, it may fail to detect silent or low-expression determinants. In contrast, gene-based detection enables identification of specific resistance determinants and mobile genetic elements, offering early warning of emerging resistance and insights into horizontal gene transfer dynamics [78,79]. Discordance between genotype and phenotype is well documented in enterococci, with resistance genes not always expressed and unexplained phenotypic resistance also occurring [80]. Therefore, integrating both approaches improves risk assessment by distinguishing current resistance from latent genetic potential, in line with recommendations from the European Food Safety Authority and the World Health Organization for comprehensive AMR monitoring frameworks [81].

In dairy ecosystems, especially during cheese production and ripening, complex microbial interactions and environmental pressures may influence both the selection and maintenance of antimicrobial-resistant enterococci. Despite increasing evidence of AMR occurrence in dairy-associated strains, the role of processing environments such as brines, equipment surfaces, and ripening chambers as reservoirs or amplification sites remains insufficiently understood [77,82]. Consequently, there is a growing need for structured surveillance frameworks capable of integrating microbial ecology, antimicrobial susceptibility, and resistome profiling across the entire cheese production chain. Current AMR surveillance systems remain fragmented and largely rely on cross-sectional sampling approaches that do not fully capture AMR evolution across processing stages [73,82]. In addition, phenotypic susceptibility testing is often not systematically integrated with molecular detection of resistance genes, limiting the ability to distinguish between expressed resistance and latent genetic potential [80]. Further studies addressing the knowledge gaps in this area are required to design efficient, effective monitorization schemes that will adequately reflect the AMR situation in cheese production chains.

## 5. Pathogens Associated with Cheese and Their Relevance to One Health

Foodborne diseases impose a heavy burden on human societies; they contribute to morbidity and mortality, strain healthcare systems, disrupt economies through lost productivity and medical expenses, and can erode consumer trust in food safety. Furthermore, the weight of foodborne disease is unequally distributed, with the most vulnerable populations bearing a heavier load. Although progress has been made to mitigate this burden, the incidence of foodborne disease is still higher than established in national developmental goals, such as Healthy People 2030 in the USA [83]. At a global scale, food safety and the prevention of foodborne illness is intrinsically linked to several of the Sustainable Development Goals for Development (SDGs) established by the United Nations, namely SDG 2—Zero Hunger, SDG 3—Good Health and Well-being, and SDG 12—Sustainable Consumption and Production. Cheese is a popular food, with the EU alone producing 10.8 million ton/year [84]. It is, in general, a safe, nutritious food. However, as any other type of food, it can harbour pathogenic bacteria and raise concerns of relevance for the One Health approach to human, animal, and environmental health. Foodborne illness is not the sole outcome of the presence of pathogens in foods and food-related environments; foodborne pathogens may also pose concerns related to AMR, virulence, and their spread. Foods and their production environments may serve as reservoir and hotspot for AMR dissemination [85]. Understanding the community structure of cheese microbiota is, accordingly, crucial for an effective control of these pathogens [86].

The main bacterial pathogens associated with cheese are *S. enterica*, *L. monocytogenes*, *Staphylococcus aureus*, and pathogenic *E. coli* (especially Shiga toxin-producing pathotypes) [87]; however, it can also vehiculate *Mycobacterium bovis* [88] and brucellae [89]. Moreover, cheese may harbour microbial toxins such as staphylococcal enterotoxins [90], biogenic amines [91], and mycotoxins [92]. Recent reviews addressing biogenic amines [91,92,93,94], staphylococcal enterotoxins [95,96], and mycotoxins [92,97] in cheese extensively cover these topics; hence a focus on relevant pathogens will be pursued hereafter.

Pathogens can reach cheeses via primary and secondary contamination. They can enter cheese using its main raw material (i.e., the cheesemaking milk) as a vehicle. Raw milk pathogenic microbiota may have different sources: dairy animals (zoonotic pathogens), milking environment and equipment, or human handlers [98,99,100,101]. To curtail their progress down the cheese production chain, pasteurization is often applied. However, some pathogens may survive this lethal unit operation, and post-thermal treatment contamination can occur, coming from the dairy plant environment [102,103]. The brine used for salting, the floors of the cheese plant, cheesemaking equipment, besides the atmosphere in the premises, maturation rooms, and cold storage equipment are possible sources of pathogens [104].

### 5.1. Staphylococcus aureus

*S. aureus* is frequently found in dairy products, as is the case of cheese. A recent study reported an incidence of 18.5% in the food samples analyzed, and 30% of the samples in which it was detected were of dairy origin [105]. It is a human pathogen of concern, which is part of the ESKAPE group. In the context of cheese, the hazard posed by *S. aureus* derives from its ability to produce enterotoxins under suitable conditions [90]. Staphylococcal enterotoxins have an emetic action on the human host and are the virulence factor implicated in staphylococcal food poisoning (SFP)—usually an acute, self-limiting foodborne disease which may vary in severity depending on the host’s susceptibility and the amount of toxin ingested [90,106]. Besides its importance in terms of public health, SFP is still one of the most important foodborne diseases from the point of view of its economic impact [106] and it is one of the most frequent health hazards associated with raw milk cheeses [107].

A staphylococcal population density above 10^5^ cfu g^−1^ is required to accumulate a sufficient dose of enterotoxins to trigger SFP. Nevertheless, controlling *S. aureus* growth in cheese may prove challenging, due to its resistance to the barriers applied during cheesemaking, as demonstrated by Câmara et al. [108,109]. However, the conditions that allow for enterotoxin production are stricter than those for growth and the presence of high numbers of staphylococci in some cheeses (e.g., Pico cheese), does not necessarily imply that enough enterotoxins have accumulated to be detectable [17].

*S. aureus* is often present in raw milk. It occurs in the milk of 20–30% of cows, and 30–40% of the milk from small ruminants [87]. It may enter raw milk due to excretion by infected animals, gain access from contaminated animal surfaces, or via cross-contamination from colonized human workers and milking equipment [109,110,111]. Human handlers have been identified as one of the main sources of, and vehicles used by *S. aureus* to access foods [112]. In raw-milk cheeses, milk is not subjected to a lethal heat treatment (pasteurization); therefore, enterotoxigenic staphylococci pose a higher risk in these cheese varieties. In cheeses made from pasteurized milk, the risk is lower but still present, because pasteurization leads to a reduction in the numbers of *S. aureus* in the milk, but it does not effectively eliminate enterotoxins accumulated prior to heat treatment [113]. Furthermore, *S. aureus* may re-enter the cheese production chain as a post-pasteurization contaminant, from human food handlers and processing equipment [107,111,114]. The ability to form biofilms on equipment surfaces enables this pathogen to survive in cheese plants and thus establish an additional reservoir from where it can disseminate throughout the cheese production chain [110].

Once it gains access to cheese, *S. aureus* finds there a mostly favourable environment. Water activity values in cheese are generally within the range that supports growth of this halotolerant pathogen. Regarding Eh, *S. aureus*, a facultatively anaerobe, may grow well both under aerobic and anaerobic conditions. However, enterotoxin production is favoured in the aerobic areas of the cheese (crust) rather than within the anaerobic matrix [114]. The main barrier that controls staphylococcal growth in cheese, preventing it from reaching the high numbers required for producing harmful enterotoxin levels, is the decrease in pH as a result of SLAB activity. If the acidification rate is insufficient, problems with enterotoxigenic staphylococci may ensue. The first hours of cheesemaking are, thus, crucial for controlling staphylococcal growth in the curd/cheese matrix [111]. The aerobic conditions and lower acidity that prevail in the crust make it more susceptible to enterotoxin accumulation than the cheese matrix [107]. Bacterial antagonism by SLAB and NSLAB is also a relevant factor that prevents enterotoxin accumulation [107,114]. The concept of using LAB as a biocontrol agent against *S. aureus* is not new [115] and has been successfully applied in cheese to reduce this pathogen’s viable numbers [109]. Another suitable barrier to ensure cheese safety is refrigeration. The lower temperature limit for *S. aureus* growth is 6 °C, while enterotoxin production is halted below 10 °C; refrigeration is, therefore, an important barrier to address the problems caused by *S. aureus* in cheese during storage. Failure in the cold chain is regarded as one of the main reasons for safety issues related to *S. aureus* growth and toxin accumulation in the cheese production chain [107]. Once ingested, *S. aureus* can even withstand inactivation by human gastrointestinal proteases [110].

*S. aureus* is a zoonotic pathogen that uses dairy products, such as cheese, as one of its dissemination routes, causing SFP, an enterotoxin-mediated foodborne intoxication, in humans. However, SFP is not the only concern posed by *S. aureus* within the scope of One Health. Although intrinsic resistance to antibiotics is deemed to be virtually absent in *S. aureus*, acquisition of genetic determinants of AMR, mainly by HGT, has endowed this pathogen with protection against important antibiotics [116]. In fact, the first description of antibiotic resistance in *S. aureus* dates back to 1942, a mere two years after the medical use of penicillin was introduced, and methicillin-resistance was observed in 1961, also two years after its introduction [117]. Resistance against other beta-lactams, glycopeptides, aminoglycosides, tetracyclines, fluoroquinolones, and macrolides (among others) has ever since been described in *S. aureus* strains [118]. Food-related *S. aureus* isolates were found to display high AMR rates against antibiotics belonging to the beta-lactam (25—80%), macrolide (33–50%), sulphonamide (49%), tetracycline (50%), and aminoglycoside (21%) classes. Resistance rates against antibiotics of the fluoroquinolone family were lower (12–13%) [85]. Furthermore, it has been demonstrated that resistance against biocides was found to promote cross-protection against antibiotics, both in hospital [119] and food [85] settings.

In *S. aureus*, genetic determinants of AMR are mainly located in mobile genetic elements (MGEs), which facilitate their dispersion by HGT. MGEs constitute ca. 15–30% of the genome of this species, making it highly variable. They can be found in genomic islands, transposons, phages, plasmids, integrative conjugative elements, and staphylococcal chromosomal cassettes. MGE transfer occurs mostly during colonization of the human host, or within biofilms [85]. Therefore, besides their role in SFP, cheese staphylococci may constitute a reservoir of genetic determinants of antimicrobial resistance (AMR) for horizontal gene exchange with other bacteria [120]. This contributes to aggravate the AMR crisis, regarded by WHO as a fundamental threat to humankind [121]. Staphylococci have been reported to trade genes such as *fexA*, *cfr*, *erm*, *tet*, *cat*, *vanA*, *bla*, *aph2″*, *aac6′*, *vgb*, *aadE*, *sat4*, and *aphA*, which confer resistance to a wide variety of antibiotics (Table 2), with other Gram-positive bacteria. HGT of *vanA* and *cfr* is of special concern, since they confer resistance to last resource antibiotics (vancomycin/teicoplanin and florfenicol/linezolid, respectively) [122].

In dairy animals, *S. aureus* is one of the main agents of contagious mastitis [123]. Therefore, mitigating the risks associated with *S. aureus* in the cheese production chain must start at the dairy farm level, with the implementation of efficient mastitis control measures [110]. Because secondary contamination is also possible with this pathogen, control must be applied at the cheese plant as well, by adhering to strict hygiene practices, controlling critical parameters, and taking advantage of starter/autochthonous LAB [109,124]. Attention must be paid to biofilms on equipment surfaces, using both hygiene-based strategies and novel surface materials that make it harder for these bacterial communities to establish themselves [85]. Asymptomatic colonization with *S. aureus* is frequent in food handlers [125] and complicates control of the “human-to-food” contamination pathway, reinforcing the need to ensure compliance to such food hygiene rules as hand washing, use of gloves, masks and hairnets. Nasal decolonization of the handlers has also been proposed [126].

**Table 2:** Resistance phenotypes resulting from presence of selected genetic determinants in staphylococci.

Genetic Determinant	Antibiotics to Which It Confers Resistance	Resistance Mechanism	Source
*fex*A	Phenicols (chloramphenicol, florfenicol)	Efflux pump (MFS transporter)	[127]
*cfr*	Phenicols, lincosamides, oxazolidinones (linezolid), pleuromutilins, streptogramin A (PhLOPS_A phenotype)	23S rRNA methyltransferase	[128]
*erm* (A/B/C/T)	Macrolides, lincosamides, streptogramin B (MLS_B phenotype)	23S rRNA methylation	[128]
*tet* (K/L/M, etc.)	Tetracyclines	Efflux (TetK/L) or ribosomal protection (TetM)	[129]
*cat* (catA/catB)	Phenicols (chloramphenicol)	Chloramphenicol acetyltransferase (enzymatic inactivation)	[130]
*van*A	Glycopeptides (vancomycin, teicoplanin)	Altered peptidoglycan target (D-Ala–D-Lac)	[131]
*bla*Z	β-lactams (penicillins)	β-lactamase production	[129]
*aph*(2″)	Aminoglycosides (gentamicin, tobramycin,	Phosphorylation (±acetylation in bifunctional enzyme)	[132]
	kanamycin)		
*aac*(6′)	Aminoglycosides	Acetylation	[129]
*vgb*	Streptogramin B	Lactonase (enzymatic inactivation)	[128]
*aad*E (ant(6)-Ia)	Aminoglycosides (streptomycin)	Adenylylation	[129]
*sat*4	Streptothricin	Acetyltransferase	[133]
*aph*A (e.g., *aph*A3)	Aminoglycosides (kanamycin, neomycin)	Phosphorylation	[134]

### 5.2. Listeria Monocytogenes

Another pathogen of concern regarding cheese is *Listeria monocytogenes*, the main species implicated in foodborne listeriosis. Listeriosis has a low incidence when compared to other foodborne diseases. Yet, in its 2024 report on listeriosis, ECDC [135] has alerted that the number of yearly listeriosis cases is on the rise. In spite of its lower incidence, listeriosis is still of concern, due to its severity, high hospitalization and fatality rates in the higher risk groups of the human population (namely, young children, elderly, pregnant women, and immunocompromised individuals). In Europe, a total of 2770 listeriosis cases were reported in 2022, the majority (67%) of which were in individuals over 64 years old. ECDC has reported an increase in the number of yearly listeriosis cases [135]. Worldwide, in 2010, there were 23 150 reported listeriosis cases in 2010, which caused over 5 thousand deaths and loss of 172 823 disability-adjusted life-years [136]. Listeriosis can assume different clinical forms: neurolisteriosis (meningitis or meningoencephalitis, 52% of human cases), neonatal/pregnancy associated listeriosis (31%), septicaemic listeriosis (14%) and, less frequently, infections in other body sites. All *L. monocytogenes* strains are considered pathogenic. They differ, nevertheless, in their virulence, with just three of the 13 serotypes accounting for 92–95% of the clinical cases [137].

Another potential threat associated with *L. monocytogenes* in cheese and cheese-related environments relates to its potential role as a reservoir of genetic determinants of AMR. Compared to other foodborne pathogens, *Listeria* strains exhibit a high rate of susceptibility to antimicrobials [138]. However, resistance to several antibiotics, from beta-lactams (penicillin G, ampicillin, oxacillin), to tetracycline, chloramphenicol, and sulphametoxazole-trimethoprim were described in listerial strains from dairy products [139]. In this pathogen, resistances to penicillin, ampicillin, and tetracycline are non-intrinsic [140]. Studies in listeriae from soil have shown that, within the *Listeria* genus, *L. monocytogenes* and closely related species carry more AMR genes than the other species [141]. Listeriae are known to engage in HGT events with other Gram-positive bacteria, e.g., enterococci, with whom they trade AMR genes such as *tet*, *erm*, *cat*, and *aad*, involved in resistance to tetracyclines, macrolides/liconsamides/streptogramins, and streptomycin, respectively [122]. The aptitude of *L. monocytogenes* to form biofilms, which are hotspots for HGT, in food-related surfaces may promote AMR trading events with other food-related bacteria [142]. Studies in soil seem to show that conjugation and transduction do not contribute substantially for HGT in listeria, but natural transformation might [143].

Listeriae are hardy bacteria, well-equipped to survive and thrive in harsh, unstable environments, such as cheesemaking facilities and cheese itself. Besides their psychrotrophic nature, listeriae also cope well with the stresses posed by acidity and salinity in cheese [137]. Seven percent of *Listeria*’s genome contains genes for adaptive regulation that enable this pathogen to face diverse environmental challenges [144]. *L. monocytogenes* carries mobile genetic elements, such as prophages and transposons, that confer considerable variability [145]. The open nature of the genome of *L. monocytogenes* provides the genetic basis for adaptation to its hosts and to a diverse range of environments [146]. *L. monocytogenes* can be found in cheeses made from both raw and pasteurized milk [147]. Dairy animals, bad quality silage, and poorly sanitized milking equipment can be a source of listeriae in the dairy farm [148], but post-thermal processing contamination is also frequent, since *L. monocytogenes* is ubiquitous in dairy environments. Dairy plants that have complex processing lines seem more prone to its presence. Its ability to form biofilms promotes persistence on dairy surfaces for periods that can go up to 10 years, despite of the cleaning and disinfection routines applied [147,149].

The main factors that affect growth/survival of *L. monocytogenes* in cheeses and cheese-related environments are temperature, moisture content, rate and extent of acidification and salting, as well as the activities of SLAB and NSLAB. However, common barriers applied in cheesemaking may not suffice to prevent listerial growth; they may even promote its survival and enhance its virulence, although the impact of cheese matrix on *L. monocytogenes* virulence is complex and strain-dependent. *L. monocytogenes* is able to survive within a wide pH range, *a*_w_ values up to 9.2, and it may grow at NaCl concentrations as high as 10% [147]. *L. monocytogenes* strains can survive HTST and LTLT pasteurization [150]. On the other hand, the high levels of milk fat in certain cheese matrices may affect its ability to colonize the host [151]. Exposure to sublethal conditions found in cheese and cheesemaking environments can provide listeriae cross-protection against other stressors (e.g., heat, salt, alkalinity, acidity, ethanol, and oxidative stress), thus reinforcing its ability to cause disease, by promoting survival unfavourable factors within the human host (viz. exposure to gastric fluids, bile, intestinal digestion products, oxidative stress within the phagosome, and competition with the intestinal microbiota). It can also impact AMR in *L. monocytogenes* [152]. Cheese SLAB and NSLAB may, however, provide an effective barrier against listeriae in cheese. Microbial consortia in raw-milk cheeses, such as St. Nectaire [153], can inhibit *L. monocytogenes*, even when pH values would otherwise permit its growth. In pasteurized-milk cheeses, it has been demonstrated that microbial consortia with a higher LAB diversity provide higher inhibitory capacity against *L. monocytogenes* [154]. LAB in cheese hold potential for controlling *L. monocytogenes*, and they have been successfully employed for that purpose in several cheese types (e.g., fresh cheese, and Gorgonzola) [155,156]. However, it must be noted that their inhibitory action is strain-dependent, depending both on the listerial and LAB strains in question [156]. LAB antilisterial activity arises from different mechanisms and compounds, niche exclusion, competitive exclusion, competition for nutrients, production of oxidizing agents, organic acids, and bacteriocin production, among others [157,158].

In *L. monocytogenes*, as in other bacterial taxa, exposure to environmental stressors may co-select for antibiotic resistance. The implicated mechanisms include cross-resistance, co-resistance, and co-regulation [159,160]. Exposure to environmental stressors such as biocides, osmotic stress, and/or oxidative stress can provide indirect selective pressure for antibiotic resistance because the corresponding genetic determinants are often simultaneously present on mobile genetic elements (e.g., plasmids, transposons, and integrons) [159,161]. Furthermore, cross-resistance mechanisms, including multidrug efflux pumps, membrane permeability changes, and global stress-response regulators, can impart tolerance to both environmental antimicrobials and clinically relevant antibiotics [162]. These mechanisms contribute to *L. monocytogenes* persistence in dairy-processing environments and may also facilitate the acquisition and dissemination of antibiotic resistance under the non-antibiotic selective pressures this bacterium encounters in cheesemaking environments. Therefore, antibiotic resistance could confer *L. monocytogenes* higher tolerance towards osmotic stress, and higher osmotic shock resistance was more frequently demonstrated in multi-resistant strains than in their counterparts that were resistant to a single antibiotic. Furthermore, exposure to high salinity provided cross-protection against antibiotics [163]. Similarly, exposure to sanitizers, such as benzalkonium chloride, led to increased resistance to cefotaxime, cephalothin, and ciprofloxacin in *L. monocytogenes* [164].

Regarding thermal and acid stresses, the results of recent works unfold a more complex picture. Antibiotic resistance was shown to impair thermal tolerance in *L. monocytogenes* [165], possibly due to the overall cost of antibiotic resistance [166]. Conversely, submitting bacteria to heat stress led to a decrease in their resistance to antibiotics. However, increased susceptibility was no longer present upon removal of thermal stresss. On the other hand, cold stress could enhance bacterial resistance to several antibiotics, and this increased resistance was not reverted once the stress was discontinued. Antibiotic-resistant strains demonstrated lower sensitivity to acid stress than their antibiotic susceptible counterparts and, when pre-adapted to an acid environment, *L. monocytogenes* strains subsequently showed higher resistance to antibiotics. However, the cross-protection effect of acid stress against antibiotics seemed to depend on the type of antibiotics used [163]. In *L. monocytogenes*, resistance to both antibiotics and dairy-related environmental stressors has been widely recognized as strain-dependent, with marked variability in tolerance to acid, osmotic, cold, and disinfectant stresses, as well as in antibiotic resistance profiles, reflecting differences in genomic content and stress-response systems among strains [166,167,168,169,170].

Although raw milk cheeses are widely regarded as presenting an elevated risk of contamination with *L. monocytogenes*, a 2018 meta-analysis across various types of European cheeses showed no significant difference in the prevalence of this pathogen in raw- and pasteurized-milk cheeses [171]. To minimize the risks associated with *Listeria* in cheese, the presence of high levels of this pathogen in the raw-materials (mainly raw milk) and intermediate products (curd, and cheese at various maturation stages) must be avoided. Pasteurization has been employed as a control measure against *L. monocytogenes* in cheese. However, since pathogen survival and post-processing contamination are possible, hygiene proves to be paramount at all steps of the cheesemaking, from primary production to storage and handling by the consumers. Hygiene practices and procedures must account and target the biofilm-producing capacity of this pathogen. Intrinsic factors in cheese may afford additional control barriers. For instance, pH values below 4.5 and *a*_w_ values below 0.92 can limit listerial growth, but this pathogen may still survive [172]. Research has shown that cheese LAB, both autochthonous and added as starters, may play important roles in controlling *L. monocytogenes* [173] in different types of cheese: fresh [155,174], soft [175], semi-soft [174], smear-ripened [174,176], semi-hard [177], and mould-ripened [156]. Screening studies indicated that only a small proportion of LAB isolates display antilisterial activity and demonstrated a high strain-to-strain variability within the same species [178,179,180].

*L. monocytogenes* can persist in dairy environments through biofilm formation, enhanced stress-response systems, and tolerance to biocides, thus increasing the risk of contamination throughout production and storage [181]. Control of *L. monocytogenes* during cheesemaking must rely on a multi-hurdle approach combining preventive hygiene, physicochemical barriers, and biological control strategies to circumvent its remarkable adaptability to individual stressors (cold, acid, salt, and biocides) that would otherwise select for tolerant or resistant subpopulations if applied alone. This reduced reliance on single intense stressors not only enhances microbial safety but also limits selective pressure that could drive antibiotic or sanitizer resistance among *L. monocytogenes* populations, thereby contributing to broader food safety and public health goals. Continuous surveillance and integrated control frameworks are therefore essential components of effective hazard management in dairy processing [158,182]. Good manufacturing practices and effective sanitation of dairy-processing environments are essential to prevent contamination and persistence, as *L. monocytogenes* can survive refrigeration, high salt concentrations, and form biofilms on equipment surfaces. Thermal treatments such as milk pasteurization remain a primary control measure, while such product characteristics as pH reduction, salt concentration, and water activity during fermentation and ripening can further limit pathogen growth. In addition, protective LAB cultures, and bacteriocins (e.g., nisin and pediocin) are increasingly used as biocontrol agents, as they produce antimicrobial compounds that inhibit *L. monocytogenes* in cheese matrices. Emerging control strategies include application of bacteriophages, protective microbial cultures, and hurdle technologies combining biological and physicochemical interventions, which can significantly reduce pathogen levels without compromising product quality [183,184,185,186].

Surface contamination of cheese with *L. monocytogenes* during its market life represents a critical food safety risk, as the rind and exterior are preferential sites for post-processing contamination and subsequent pathogen proliferation during storage and ripening; targeted surface control is therefore essential to prevent foodborne listeriosis associated with ready-to-eat cheeses. Recent studies demonstrate that applying antimicrobial edible films and coatings, such as chitosan-based matrices incorporated with zinc oxide nanoparticles [187] or bacteriocin-containing whey protein films [188], can significantly reduce *L. monocytogenes* counts on cheese surfaces and inhibit migration into the cheese matrix, thus offering a food-grade, bioactive barrier that complements traditional hygiene measures and processing controls.

### 5.3. Salmonella enterica

*Salmonella enterica* remains a relevant, albeit relatively less frequent hazard in cheese compared to other pathogens, with sporadic outbreaks still reported. Prevalence studies generally indicate low detection rates in finished cheeses, often below 1–6%, which reflect the effectiveness of hygiene strategies implemented in cheesemaking, as well as the inhibitory effects of fermentation and ripening conditions (e.g., low pH, or decreasing *a*_w_) [189,190]. However, the risk is higher for cheeses produced from raw milk, where contamination can originate from the raw material. Outbreaks associated with raw-milk dairy products continue to be documented, although pasteurization substantially reduces the likelihood of *Salmonella* survival and transmission [191], by effectively inactivating this pathogen [192]. *Salmonella* presence in cheeses made from pasteurized milk is frequently attributed either to post-process contamination [190] or to human handling [193].

The survival of salmonellae during cheesemaking is strongly influenced by the combined effect of several intrinsic and extrinsic environmental factors. Acidification during fermentation, driven by LAB, lowers the pH of the curd and is one of the most important inhibitory hurdles; in fact, organic acid accumulation and the associated decrease in intracellular pH reduce the growth potential of salmonellae [194]. During ripening, progressive decreases in *a*_w_ due to moisture loss and salt diffusion further limit survival by restricting metabolic activity [194]. Cheese Eh, which becomes more reduced as microbial metabolism progresses, together with temperature regimes during fermentation, ripening, and refrigerated storage, also influence pathogen behaviour, with lower temperatures generally restricting growth but allowing prolonged survival [194]. In addition, LAB contribute other inhibitory metabolites besides the organic acids resulting from their fermentative metabolism, such as H_2_O_2_, and bacteriocins, and compete for nutrients, thus creating ecological pressure that can trigger inhibition or inactivation of *Salmonella* in many cheese varieties [195]. Milk pasteurization provides a useful hurdle, since it effectively inactivates this pathogen [192]. Consequently, the combined action of acidification, reduced *a*_w_, temperature control/thermal treatments, and LAB-derived antimicrobial compounds largely determines the fate of *S. enterica* in cheese matrices [20,194,196,197]; this provides evidence of the importance of process control toward mitigation of the risks it poses.

Although *Salmonella enterica* is less frequently detected in cheese than other foodborne pathogens, it remains relevant from a One Health perspective because dairy products, particularly those produced from raw milk, can act as vehicles that link animal reservoirs, food systems, and human infections [191,198]. In addition, the emergence and dissemination of antimicrobial-resistant *Salmonella* across livestock, food, and human populations highlight the interconnected nature of antimicrobial use in agriculture and public health, thus emphasizing the need for integrated surveillance and control strategies across the food chain [199,200].

Antibiotic resistance among *Salmonella enterica* isolates recovered from cheese and dairy products constitutes a food safety and public health concern, particularly in regions where raw milk and traditional cheeses are widely consumed. Multidrug-resistant *Salmonella* has been repeatedly detected in retail dairy products such as soft cheeses and raw milk. Several serotypes exhibit resistance to multiple antibiotic classes including erythromycin, oxacillin, nalidixic acid, and other clinically important agents, which demonstrates the potential risk of transmission of resistant strains through contaminated foods [201]. Salmonellae isolated from traditional dairy products frequently show resistance to tetracycline and other commonly used antibiotics, thus reflecting the selective pressure associated with antibiotic use in livestock [202]. Surveillance along the entire milk supply chains has demonstrated that antimicrobial-resistant salmonellae are widespread in milk, milking equipment, and hand swabs from handlers; this justifies the importance of hygiene and monitoring throughout the dairy processing sector to reduce dissemination of resistant bacteria [203]. Strengthened antimicrobial stewardship in animal husbandry, rigorous control in the cheesemaking processes, and improved surveillance are accordingly required to mitigate the impact of antibiotic resistance of *Salmonella* in the dairy value chain [200], making it a challenge that is best tackled by an integrated, multidisciplinary, cross-sectorial approach, such as the one provided by the One Health framework.

### 5.4. Enteropathogenic E. coli Strains

Pathogenic *E. coli* (especially Shiga toxin-producing *E. coli*, STEC) can use the dairy chain to circulate between livestock reservoirs, food production environments, and humans [204,205]. As with *Salmonella*, surveys generally report higher prevalence in raw-milk cheeses than in pasteurized-milk ones, with studies detecting STEC in ca. 7.3% of Alpine raw-milk cheeses [206], while prevalence in pasteurized-milk cheeses is typically much lower, due to the lethality of the thermal treatment employed [204]. Contamination may originate from fecal shedding by ruminants, raw milk, water, equipment, or handlers during cheesemaking; this emphasizes the role of farm-to-food environmental interfaces in pathogen dissemination [205]. Due to the multiplicity of sources and contamination routes, an integrated strategy, based on the One Health approach, is urgent to mitigate the risks associated with the presence of STEC in cheeses. During cheesemaking, the fate of *E. coli* is influenced by acidification, decline in *a*_w_, Eh changes, temperature regimes during curd cooking (if used), ripening and storage conditions, as well as microbial competition, which can collectively inhibit its growth. However, survival to these barriers is still possible, under favourable conditions [204]. Additionally, SLAB and NSLAB contribute inhibitory compounds such as organic acids, H_2_O_2_, and bacteriocins, which create additional hurdles that limit persistence of pathogenic *E. coli* in cheese matrices [204].

As with the previously discussed pathogens, cheese can act as a potential vehicle for the dissemination of antibiotic-resistant *E. coli* along dairy chains, particularly when produced from raw milk or under suboptimal hygienic conditions [205,207]. Several studies have reported occurrence of multidrug-resistant *E. coli* in cheeses and other dairy products, including strains carrying resistance to β-lactams, tetracyclines, and sulphonamides. This reflects antimicrobial selection pressures in livestock production and demonstrates the role of dairy foods as potential transmission routes for AMR strains [207,208]. In addition, cheese microbiota is prone to dissemination of antimicrobial resistance genes (ARGs), since it may bear plasmids, integrons, and other mobile genetic elements carrying such determinants as *bla*, *tet*, or *sul* genes, that can be exchanged between bacteria within food matrices or during passage through the human gastrointestinal tract [208,209]. Consequently, surveillance of antimicrobial-resistant *E. coli* and associated ARGs in dairy products is important for integrated monitoring of antimicrobial resistance across the animal-food-human interface, in compliance with the One Health principles [207,209].

To prevent dissemination of ETEC, their virulence factors and ARGs, it is important to control their entry, dissemination and growth in the cheese production chain. Control of this pathogen relies primarily on preventive measures at farm and processing levels, which involve hygienic milking practices, effective sanitation of equipment, and use of milk pasteurization whenever the production protocols allow, to substantially reduce the risk of transmission from contaminated raw milk to cheese [205,210]. As with other cheeseborne pathogens, the intrinsic hurdles applied during cheesemaking can limit the growth and survival of *E. coli*, particularly when manufacturing parameters are carefully controlled as part of validated production processes [204,206]. Protective LAB cultures can similarly inhibit pathogenic *E. coli* via competitive exclusion and the multiple aspects of their antimicrobial activity, providing a promising biocontrol strategy in dairy products [21,51]. Finally, emerging approaches such as bacteriophage application and antimicrobial packaging technologies are under scrutiny as alternatives to reduce the risks of contamination at the post-processing, storage and distribution stages of cheesemaking [51,211].

## 6. Cheese and Its Production Chain as Reservoirs/Pathways for Beneficial Microbes

Cheese and its production chain constitute complex microbial ecosystems that can act as reservoirs and transmission routes for both pathogenic and beneficial microorganisms, reflecting the diverse microbiota originating from milk, processing environment, and starter or adjunct cultures [28,29]. Cheeses harbour technologically and functionally important microorganisms, including LAB, that contribute to fermentation, flavour development, and microbial stability [29,212]. These microorganisms may constitute a valuable source of beneficial microbes bearing technological and probiotic potential [29,212].

Cheese microbiota encompasses a range of beneficial microorganisms that contribute to flavour development, texture formation, and convey potential health benefits. LAB predominate among these, driving primary acidification and producing metabolites that enhance safety and sensory qualities [212,213]. To date, 16 LAB genera have been described in cheese, belonging to the families *Enterococcaceae* (*Bavariicoccus*, *Enterococcus*, *Tetragenococcus*, *Vagococcus*), *Lactobacillaceae* (*Companilactobacillus*, *Lactobacillus, Lacticaseibacillus*, *Lactiplantibacillus*, *Latilactobacillus*, *Lentilactobacillus*, *Leuconostoc, Levilactobacillus*, *Pediococcus*, *Weissella*), and *Streptococcaceae* (*Lactococcus*, *Streptococcus*) [59,214,215,216]. Their main roles are summarized in Table 3.

Some LAB strains isolated from cheeses exhibit probiotic properties, suggesting potential health benefits beyond their traditional fermentation roles. They can limit pathogen colonization at gastrointestinal level due to their antimicrobial properties and/or competitive exclusion [59], modulate host immunity [59,217,218], contribute to regulate cholesterol levels [219,220], release opioid-like peptides such β-casomorphins from casein [221], and modulate the gut–brain-skin axis [222]. Cheese-derived LAB, such as strains of *Lacticaseibacillus paracasei*, *Lacticaseibacillus rhamnosus*, and *Lactiplantibacillus plantarum* have demonstrated their ability to assimilate cholesterol in vitro, under simulated gastrointestinal conditions [223,224,225]. The mechanisms implicated in LAB action on cholesterol involve binding of this molecule to the bacterial cell surface, incorporation into cell membranes, or reducing its solubility through bile salt hydrolase-mediated micellar effects [217,226]. In animal models, administration of LAB strains isolated from cheese has been associated with lower serum cholesterol and improved lipid profiles [227], likely mediated by bile salt activity, modulation of lipid metabolism, and altered bile acid reabsorption [228]. In humans, although direct clinical trials specifically using cheese-associated LAB are limited, some probiotic interventions with dairy LAB (including strains found in cheeses) have produced modest reductions in total and LDL-cholesterol in hypercholesterolaemic subjects [229,230,231]. Collectively, these studies indicate that cheese-derived LAB can influence cholesterol metabolism both in vitro and in vivo, though effects are strain-specific and influenced by host factors and dietary context.

Cheese-derived lactic acid bacteria contribute to human health not only through their traditional fermentation roles but also by producing bioactive peptides, including casein-derived sequences with opioid-like activity, which have been identified both in vitro and during cheese ripening; however, direct analgesic effects in humans remain unconfirmed [232,233]. Certain GABA-producing LAB strains isolated from cheese starters have demonstrated antidepressant-like effects in preclinical models, reducing depression-related behaviours via modulation of the gut–brain axis [234,235,236]. Systematic reviews and meta-analyses of probiotics in humans, including dairy-associated *Lactobacillus* and *Bifidobacterium* strains, have reported reductions in depressive symptoms and some improvements in mood, highlighting the relevance of fermented foods such as cheese as vehicles for psychobiotic strains [237]. These findings demonstrate the potential of cheese microbiota as reservoirs of microbial metabolites that impact neurophysiological processes and, accordingly, provide interesting avenues for research. However, clinical evidence for some outcomes remains preliminary.

The cheese production chain provides a pathway for dissemination of a diverse array of beneficial microorganisms, particularly LAB, that can act beyond the immediate food matrix by interacting with human, animal, and environmental microbiomes [27,28]. LAB strains associated with cheese have been shown to survive transit through the gastrointestinal tract and modulate host gut microbiota composition, thus potentially influencing metabolic, immunological, and barrier functions, central to human health outcomes in a One Health framework [59,217]. LAB can also compete with or inhibit pathogens in food and in the gut via production of organic acids, bacteriocins, and other antimicrobials, thereby contributing to both food safety and reduced pathogen dissemination [238,239,240]. Furthermore, cheese microbiota can reflect and influence wider microbial exchanges between livestock, processing environments, and humans, which illustrates how foodborne beneficial microbes are part of interconnected microbial networks that span agricultural, environmental, and clinical settings [29,212]. The movement of beneficial microorganisms through cheese and its production systems has multidimensional impacts on microbial ecology, food systems, and host health that align with One Health principles and emphasize the interdependence of human, animal, and ecosystem wellbeing. Although the One Health approach has been applied to mitigate the challenges associated with the spread of pathogens and their genetic determinants of virulence and AMR, applying is principles to the dissemination of beneficial microbes through fermented foods, such as cheese, has been proposed and could provide a useful framework to promote healthy, sustainable food systems [241].

## 7. Cheese and the Antimicrobial Resistance Crisis—A One Health Perspective

### 7.1. One Health and Multisectoral Collaboration in Facing the Emergence of Antimicrobial Resistance

In recent years, the One Health approach has been widely recognized in scientific research as a fundamental strategy to address several global health challenges, including antimicrobial resistance (AMR). This interdisciplinary and holistic concept views human and animal health as interdependent and closely connected to the ecosystems in which they coexist [242]. Major international organizations, such as the World Health Organization (WHO), the World Bank, the International Monetary Fund (IMF), and the G8, have officially acknowledged AMR as one of the greatest public health threats of the 21st century [243].

Antimicrobials, including antibiotics, antivirals, and antifungals, are essential for treating infections in humans and animals, as well as for agricultural applications. However, resistance arises when microorganisms undergo genetic changes that render them insensitive to the drugs designed to eliminate [244]. The inappropriate use of these agents, such as administering antibiotics for viral infections (e.g., influenza) or using them as growth promoters in livestock production, accelerates the development of resistance. As a result, the world is facing a growing shortage of effective antimicrobial therapies, which threatens decades of progress in medicine and public health [245].

The One Health approach is vital in the fight against AMR because it fosters coordinated action across multiple sectors—human, veterinary, environmental, and agricultural, at various levels of society. The environment and food systems are recognized as significant pathways for transmission of resistant pathogens between humans and animals. Although the emergence of AMR in the environment is well acknowledged, research aimed at better understanding its mechanisms and transmission dynamics is still ongoing [246]. Access to consolidated and timely data on antimicrobial use and AMR in communities, healthcare facilities, food production systems, and environmental settings is essential to design and implement effective mitigation strategies [76]. By mobilizing various disciplines and communities, the One Health approach offers an integrated and effective response to this global threat. It is indeed crucial to preserve the efficacy of antimicrobial agents and ensuring the long-term sustainability of both public and animal health systems.

### 7.2. Microbial Contamination in Dairy and One Health Strategies to Contain Spread of Resistance

**Figure 6:**
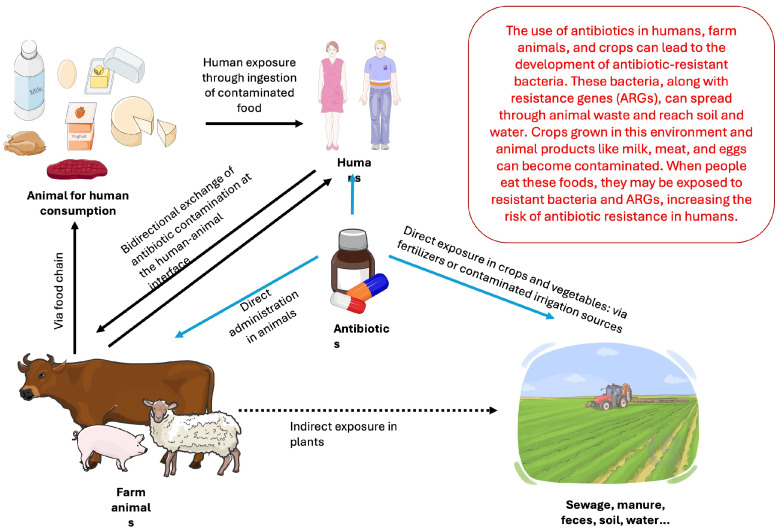
Diagrammatic representation of the routes of transmission of AMR between farm animals, the wider environment and humans.

Cheese, particularly those produced from raw milk or through non-standard artisanal practices, represents a significant vector for dissemination of antibiotic resistance genes (ARGs) (Figure 6) [66]. Both pathogenic and non-pathogenic commensal bacteria have been isolated from fermented dairy products, where they not only persist but also bear the potential to exchange mobile genetic elements with other bacterial species [247,248,249]. This feature constitutes a growing concern within the context of public health and the One Health framework.

Table 4 summarizes the AMR genes that have been described in pathogens associated with cheese and dairy settings. Among the bacterial genera most frequently associated with ARGs in cheese, *Enterococcus* spp. and *Staphylococcus* spp. are of particular importance. Species such as *Enterococcus faecalis* and *Enterococcus faecium* are commonly isolated from ripened cheeses made from raw milk, and exhibit multidrug resistance, particularly to tetracyclines and macrolides, mediated by genes such as *tet*(M), *tet*(L), and *erm*(B). These genetic determinants are often located in plasmids and transposons, which facilitate their interspecies transfer [250].

Furthermore, strains of *S. aureus*, *Staph. epidermidis*, and *Staph. equorum*, also isolated from artisanal cheeses, have been shown to harbour resistance genes such as *cat* (chloramphenicol resistance) and *msr*(A) (macrolide resistance). Notably, experimental studies have demonstrated the conjugative transfer of these genes to *S. aureus*, thereby increasing the risk of generating highly resistant strains with clinical relevance [262]. In Brazilian dairy products, the presence of *S. aureus* carrying the *mec*A (methicillin resistance) and *bla*Z (penicillin resistance) genes has been confirmed, even in samples that had undergone inadequate pasteurization [263].

Enteric pathogens such as *L. monocytogenes* and *S. enterica* subsp. *enterica* serovar Typhi have also been detected in artisanal cheeses using quantitative PCR methods, which offer higher sensitivity than conventional culture-based techniques. These microorganisms, in addition to their intrinsic pathogenic potential, may act as reservoirs for ARGs, thereby increasing the risk of foodborne outbreaks involving multidrug-resistant strains [264,265,266].

Bacterial contamination with resistance potential can occur at multiple stages throughout the cheese production chain. Raw milk serves as a primary vehicle for resistant microorganisms, including *Enterococcus* and *Staphylococcus*, whose ARGs can persist even after insufficient thermal processing [266,267,268,269]. An additional critical entry point is the use of contaminated starter cultures, such as *Streptococcus thermophilus* strains harbouring *tet*(K), which can serve as reservoirs of resistance genes [270,271]. Inadequate sanitation of processing equipment, and exposure to environmental sources during the ripening process can further contribute to introduce resistant bacteria, particularly in non-industrial or artisanal settings where controls are often less stringent.

A particularly concerning aspect is the potential for horizontal gene transfer (HGT) between bacteria present in cheese, and those within the human gut microbiome following consumption. Simulated gastrointestinal tract models have demonstrated transfer of *tet*(M) from *E. faecium* to *Lactobacillus* spp., thus highlighting the role of commensal bacteria as intermediaries in the dissemination of ARGs to opportunistic pathogens [272].

From a clinical standpoint, consumption of cheeses contaminated with resistant strains, such as methicillin-resistant *S. aureus* (MRSA) or vancomycin-resistant *Enterococcus* (VRE), poses a significant risk, especially for immunocompromised, elderly, or hospitalized individuals [273,274,275,276]. The ability of these organisms to colonize the gastrointestinal tract, and subsequently cause systemic infections underscores the role of food products as underrecognized vehicles for antimicrobial resistance transmission.

Therefore, controlling the spread of ARGs in dairy products requires an integrated and multifaceted approach. This includes strict milk pasteurization protocols, careful selection and monitoring of starter cultures to ensure absence of ARGs, rigorous hygiene practices throughout the production process, and continuous molecular surveillance of microbial communities in dairy products. Additionally, regulatory frameworks and public education campaigns are essential to mitigate the risks associated with consumption of unregulated artisanal cheeses, and to prevent further spread of antimicrobial resistance throughout the food chain.

## 8. Conclusions

Raw materials (milk), unit operations applied, and hygiene conditions prevailing during cheesemaking contribute to shape the unique microbiota of cheeses. In this product, LAB (SLAB and NSLAB) predominate, but pathogens can still be present. Cheeses and their production chains provide reservoirs and pathways for dissemination of LAB with beneficial effects upon consumers’ health, but can also contribute to spread pathogens, genetic determinants of virulence, and ARGs. Both LAB and pathogens can function as reservoirs for genes that can confer virulence or antibiotic resistance. Due to their propensity to engage in recombination events, enterococci are of particular interest in this regard and could be used as sentinel microorganisms for the surveillance of ARGs in cheese-associated ecosystems. The present knowledge on the impact of unit operations implicated in cheesemaking upon cheese microbiome and resistome emphasizes the importance of process validation and strict control to ensure the safety of this widely consumed dairy product within the One Health continuum. Thus, ensuring the microbiological safety of cheese requires a One Health approach that integrates milk production, cheesemaking practices, environmental hygiene, and antimicrobial resistance surveillance to safeguard public, animal, and ecosystem health [129].

## Figures and Tables

**Table 1:** Cheese consumption and production in different regions of the World.

Region	Estimated per Capita Consumption (kg/Year)	Production (10^6^ Tonnes/Year)
Europe (including the EU)	15–20.6 [1,2]	10.5–11.0 [2,3]
North America	15–18 [1,4]	6.0–6.5 [2]
Oceania	10–15 [1]	1.0 [2]
Latin America	5–10 [1]	2.5–3.0 [2]
Near East and North Africa	3–6 [1]	1.0 [2]
Asia	1–5 [1,4]	2.0–2.5 [2]
Sub-Saharan Africa	<1–2 [1]	0.7–1.0 [2]
World total	2.5–3.0 [5]	22.6 [2]

**Table 3:** LAB genera described in cheese and their roles.

Genus	Roles	Source
*Lactococcus*	Primary starter culture; acidification, texture/flavour development; some strains with probiotic potential	[214]
*Lactobacillus*	NSLAB; ripening, flavour, aroma; includes genera reclassified from *Lactobacillus*; some strains with probiotic potential	[214]
*Lacticaseibacillus*	NSLAB; ripening, proteolysis; many strains described as potential probiotics	[59]
*Lactiplantibacillus*	NSLAB; flavour and aroma formation; includes strains with documented probiotic properties	[59]
*Latilactobacillus*	NSLAB; carbohydrate metabolism during ripening; potential probiotic strains reported	[59]
*Levilactobacillus*	NSLAB; heterofermentative; contributes to flavour; some strains show probiotic potential	[59]
*Lentilactobacillus*	NSLAB; ripening, flavour, histamine production in some cases; probiotic potential strain-dependent	[59]
*Companilactobacillus*	Minor NSLAB; contributes to microbiota diversity; occasional probiotic characterization	[215]
*Leuconostoc*	Starter adjunct or NSLAB; contributes diacetyl, acetoin, CO_2_, texture; some strains with probiotic traits	[214]
*Enterococcus*	NSLAB; proteolysis, flavour; some strains used as probiotics, but safety depends on strain	[215]
*Pediococcus*	NSLAB; contributes flavour, texture; limited reports of probiotic potential	[215]
*Weissella*	NSLAB; heterofermentative; contributes aroma, EPS, texture; some probiotic strains reported	[214]
*Tetragenococcus*	Halotolerant NSLAB; detected in high-salt/brined cheeses; may contribute to proteolysis	[216]
*Bavariicoccus*	Minor genus detected in artisanal cheeses; role not fully characterized	[215]

**Table 4:** AMR genes reported in relevant dairy- and cheese-associated pathogens.

Bacterial Taxon	AMR Genes	Confirmed Resistance	Source Context	References
*S. aureus*	*cat*, *msr(A)*, *mecA*, *blaZ*, *tet(K)*	Chloramphenicol, Macrolides, Methicillin, Penicillin, Tetracyclines	Artisanal cheeses (Brazil), MRSA reported in dairy matrices	[251]
*S. aureus*	*mepA*, *tet(K)*, *tet(O)*, *ermB*, *aad(6)*, *smr*, *linA*, *aph(3′)-IIIa*	Tetracyclines, Macrolides, Aminoglycosides, Lincosamides, Multidrug efflux	Studies in cheeses from China and Brazil showing MDR and efflux systems	[252]
*E. coli*	*mphA*, *aadA*, *dfrA1*, *intI1*	Macrolides, Aminoglycosides, Trimethoprim, Integrons	Foodborne isolates with mobile genetic elements (MGEs)	[253]
*E. coli*	*bla_TEM_*, *bla_CMY_*_-*2*_, *bla_CTX-M_*, *tet(A)*, *tet(B)*, *mphA*, *aadA*	Beta-lactams (including extended spectrum), Tetracyclines, Macrolides, Aminoglycosides	Raw milk cheeses; ESBL-producing *E. coli* in dairy farms	[254,255]
*L. monocytogenes*	*tet(M)*, *tet(S)*, *ermB*, *cat*, *aad*	Tetracyclines, Macrolides, Chloramphenicol, Streptomycin	Horizontal gene transfer from *Enterococcus* spp. in dairy environments	[256]
*L. monocytogenes*	*penA*, *fosX*, *lde*, *mdrL*	Beta-lactams (reduced susceptibility), Fosfomycin, Efflux-mediated MDR	Genes intrinsic + acquired resistance in food isolates	[257]
*Salmonella* spp.	*gyrA* (mutations), *parC*, *intI1*	Fluoroquinolones, Quinolones, MDR via integrons	Genomic studies of foodborne MDR strains	[258,259]
*Salmonella* spp.	*bla_TEM_*, *bla_CMY_*_-*2*_, *tet(A)*, *tet(B)*, *sul1*, *qnrB19*, *floR*, *aadA2*	Tetracyclines, Beta-lactams, Sulfonamides, Quinolones, Florfenicol, Aminoglycosides	Dairy-associated strains and livestock reservoirs	[260,261]

## Data Availability

No new data were created or analyzed in this study. Data sharing is not applicable to this article.
